# Supplementary material for: Evaluating spatially variable gene detection methods for spatial transcriptomics data
Source: Genome Biol. 2024 Jan 15;25:18. doi: 10.1186/s13059-023-03145-y (PMC10789051; doi:10.1186/s13059-023-03145-y)
Supplement: Supplementary file 1 — Additional file 1: Fig. S1. Summary information and statistics of the spatial transcriptomics datasets used for evaluating concordance, statistical significance, and reproducibility of SVG detection methods in this study. Fig. S2. Pairwise correlation of SVG rankings reported by each method for individual spatial transcriptomics datasets. Fig. S3. Comparison of different gene statistics to rank SVGs. Fig. S4. (a-b) Boxplot of correlations of SVG rankings reported by each method against all other methods. Each dot denotes the results for a spatial transcriptomics dataset. The dots are coloured (a) by the total number of spatial spots in the dataset or (b) by the spatial technology platform. (c) Bar plot denoting the number of statistically significant SVGs reported by each method for each spatial transcriptomics dataset. An adjusted p-value threshold of 0.05 reported by each method for each dataset. Fig. S5. Heatmaps of the overlap of SVGs reported by each method for each spatial transcriptomics dataset. Fig. S6. Relationship between SVG statistics and proportion of zero of genes. Fig. S7. Simulation of spatial transcriptomics data. Fig. S8. Spatial patterns of spatially variant genes. Fig. S9. Spatial patterns of spatially invariant genes. Fig. S10. ROC curves of spatially variable gene detection. Fig. S11. Performance of SVGs selected by each SVG method for clustering spatial domains in the mouse embryo. [file 13059_2023_3145_MOESM1_ESM.pdf]

Supplementary figures

a

Reference and platform	Species	Tissue	# Location	# Gene	Source
Liu et al., DBiT-seq, D1	Mouse	Embryo E12	1,615	12,591	GSE137986
Liu et al., DBiT-seq, D2	Mouse	Embryo E11	1,640	12,197	GSE137986
Xia et al., MERFISH	Human	Osteosarcoma	645	12,903	Supplementary file
Eng et al., seqFISH+	Mouse	Brain cortex	524	10,000	Zenodo#2669683
Rodriques et al., Slide-seqV1	Mouse	Cerebellum	1,574	7,885	Broad Institute
Marshall et al., Slide-seqV2	Human	Kidney cortex	8,794	12,474	CELLxGENE Discover
Stickels et al., Slide-seqV2	Mouse	Hippocampus	3,870	10,658	Broad Institute
Vickovic et al., SM-Omics, D1	Mouse	Brain cortex	830	8,990	Broad Institute
Vickovic et al., SM-Omics, D2	Mouse	Brain cortex	707	6,404	Broad Institute
Ji et al., ST	Human	Squamous carcinoma	1,140	17,823	GSM4284322
Navarro et al., ST	Mouse	Hippocampus	602	18,008	Mendeley#6s959w2zyr.1
Biancalani et al., Visium	Mouse	Primary motor cortex	2,177	31,053	Google Cloud
Ferreira et al., Visium, D1	Mouse	Kidney sham	1,833	13,586	GSE171406
Ferreira et al., Visium, D2	Mouse	Kidney ischemia	2,050	13,734	GSE171406
Hunter et al., Visium	Zebrafish	Melanoma	1,548	32,268	GSE159709
Janosevic et al., Visium	Mouse	Kidney sepsis	1,888	13,710	GSE154107
Joglekar et al., Visium	Mouse	Prefrontal cortex	3,017	15,440	GSE158450
Lopez et al., Visium, D1	Mouse	Lymph node	369	13,948	GSE173776
Lopez et al., Visium, D2	Mouse	MCA205 tumour	2,125	15,976	GSE173773
McCray et al., Visium	Human	Prostate	722	36,601	GSM4837767
Wu et al., Visium, D1	Human	Breast cancer	4,781	15,325	Zenodo#4739739
Wu et al., Visium, D2	Human	Breast cancer	4,821	15,901	Zenodo#4739739

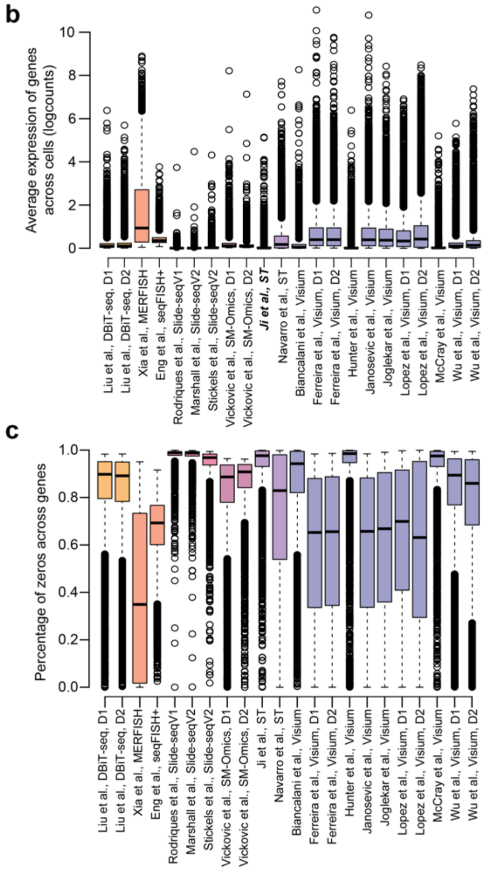

Fig. S1. Summary information and statistics of the spatial transcriptomics datasets used for evaluating concordance, statistical significance, and reproducibility of SVG detection methods in this study. (**a**) Dataset reference table, summarising basic information and source of each dataset. information. (**b, c**) Boxplot of mean gene expression and proportion of zero of gene across cells in each dataset.

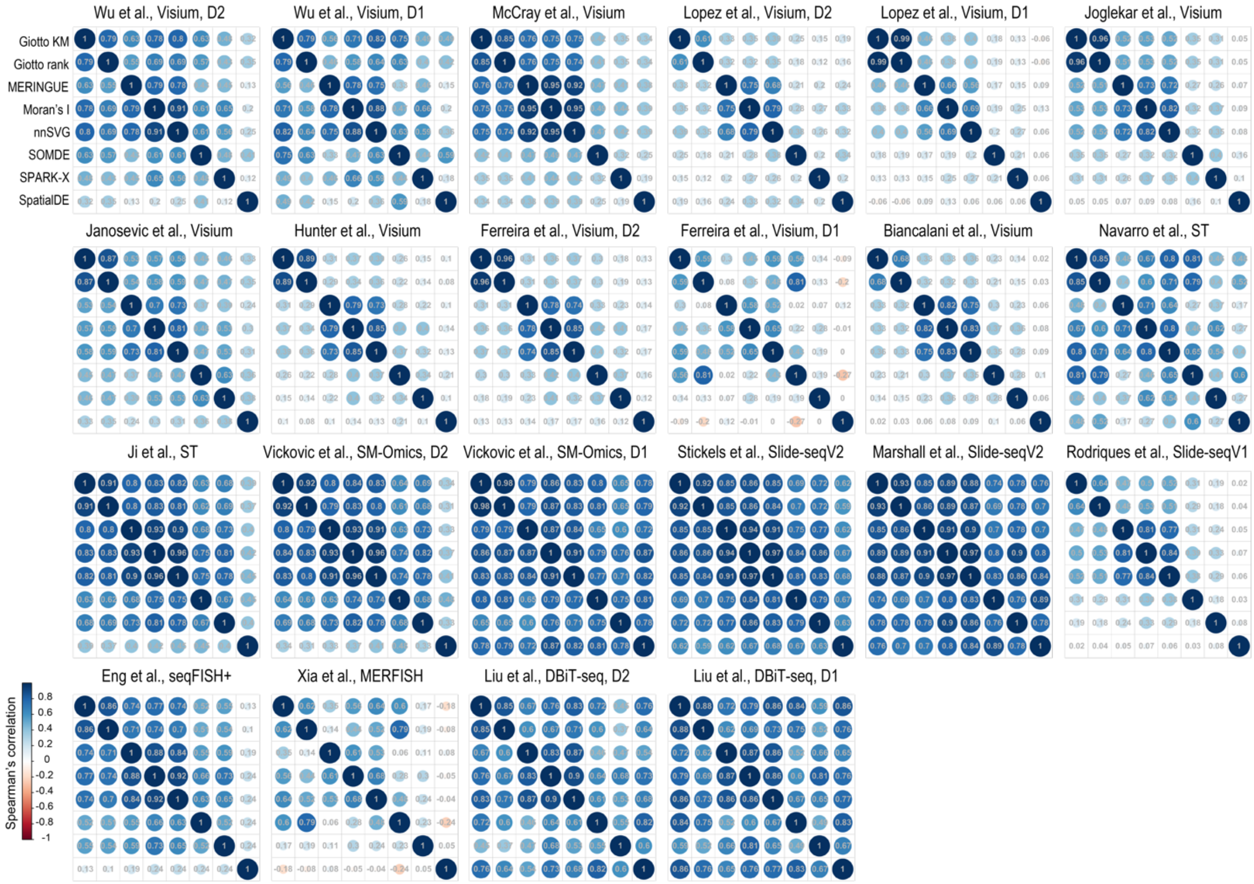

Fig. S2. Pairwise correlation of SVG rankings reported by each method for individual spatial transcriptomics datasets.

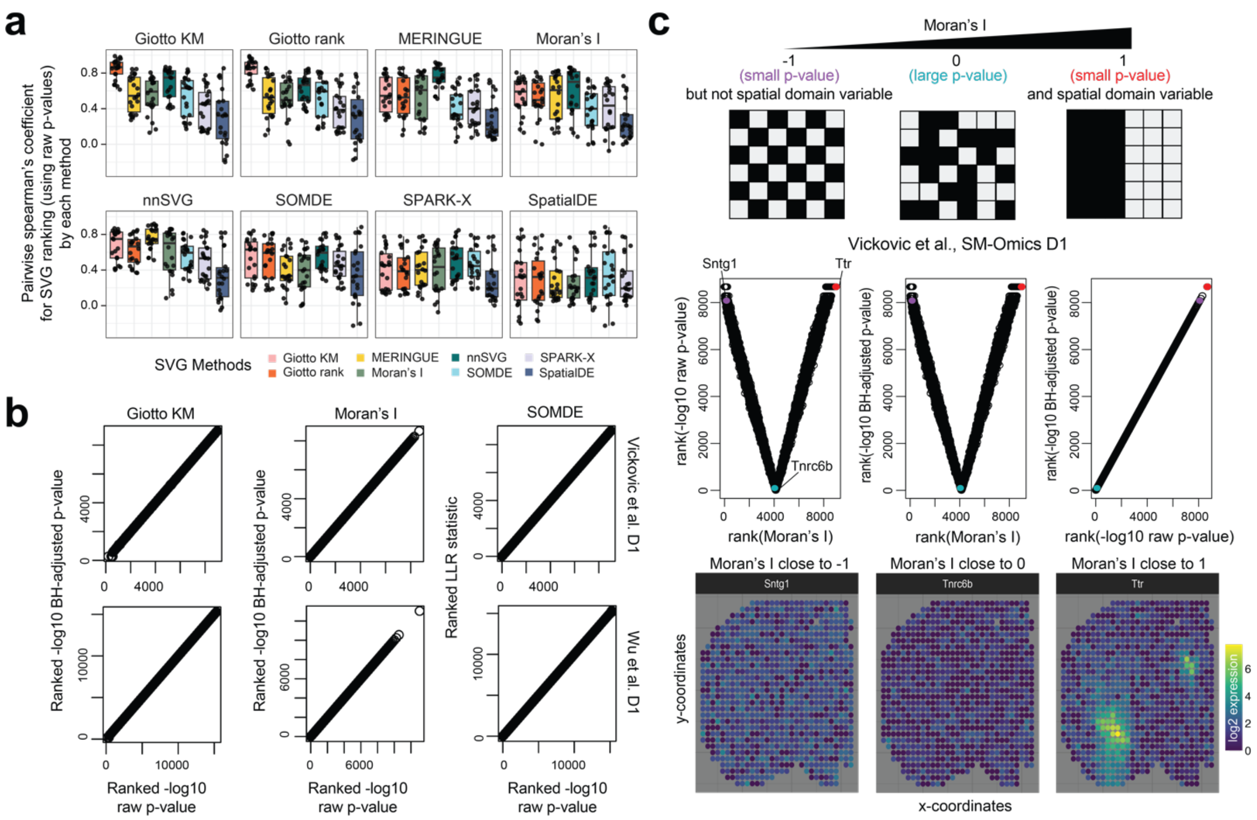

Fig. S3. Comparison of different gene statistics to rank SVGs. (**a**) Concordance of SVG rankings using raw-pvalues reported from each SVG detection method. (**b**) SVG statistics from Giotto KM, Moran's I and SOMDE from two representative datasets compared against their transformed raw-pvalues. (**c**) Top, Schematic of Moran's I spatial autocorrelation. Middle, Scatterplots of ranked Moran's I statistic versus transformed raw p-values and BH-adjusted p-values with three genes representing distinct spatial relationships highlighted an example dataset. Bottom, Visualisation of the gene expression pattern of the three representative genes.

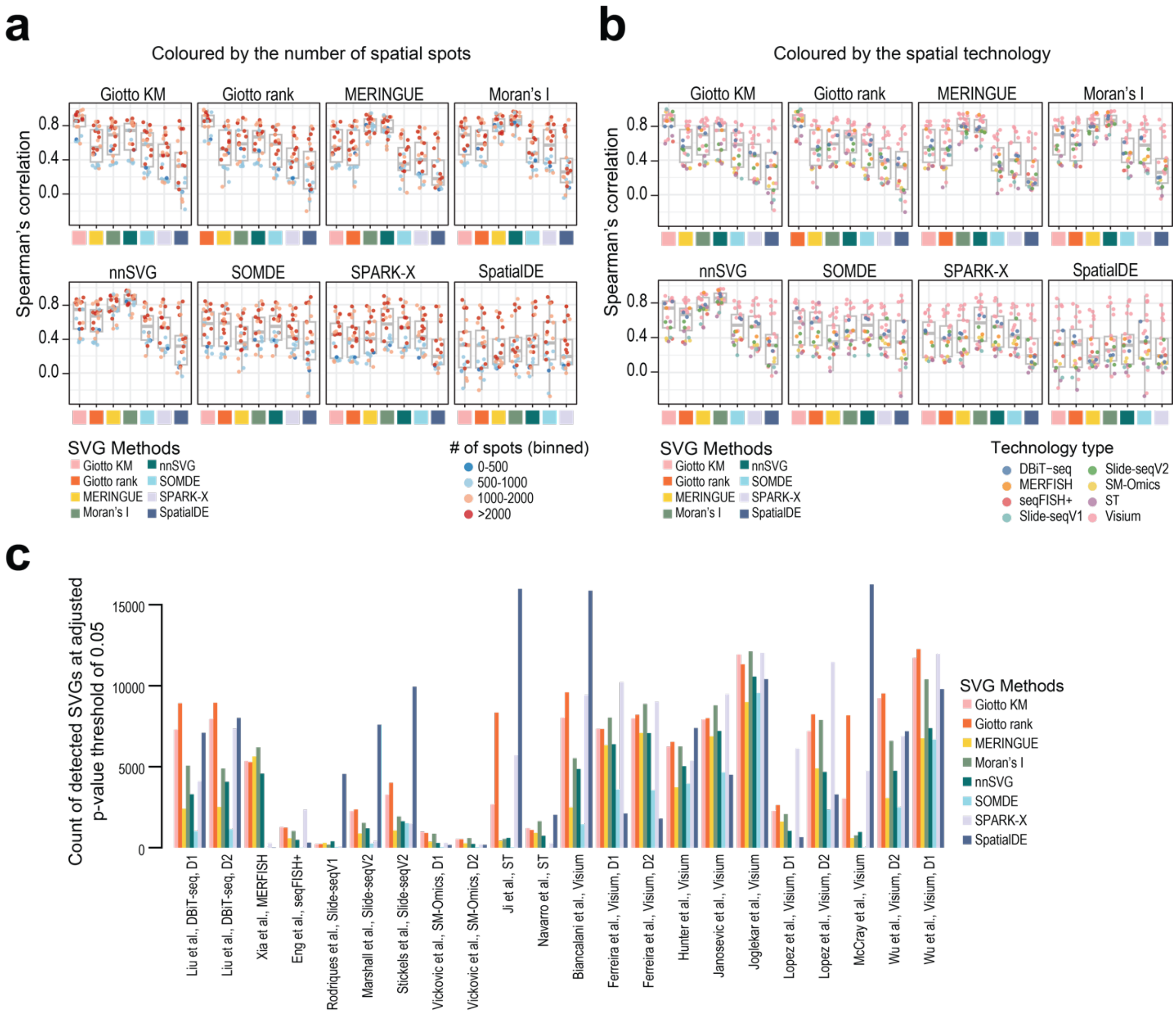

Fig. S4. (**a-b**) Boxplot of correlations of SVG rankings reported by each method against all other methods. Each dot denotes the results for a spatial transcriptomics dataset. The dots are coloured (a) by the total number of spatial spots in the dataset or (b) by the spatial technology platform. (**c**) Bar plot denoting the number of statistically significant SVGs reported by each method for each spatial transcriptomics dataset. An adjusted p-value threshold of 0.05 reported by each method for each dataset.

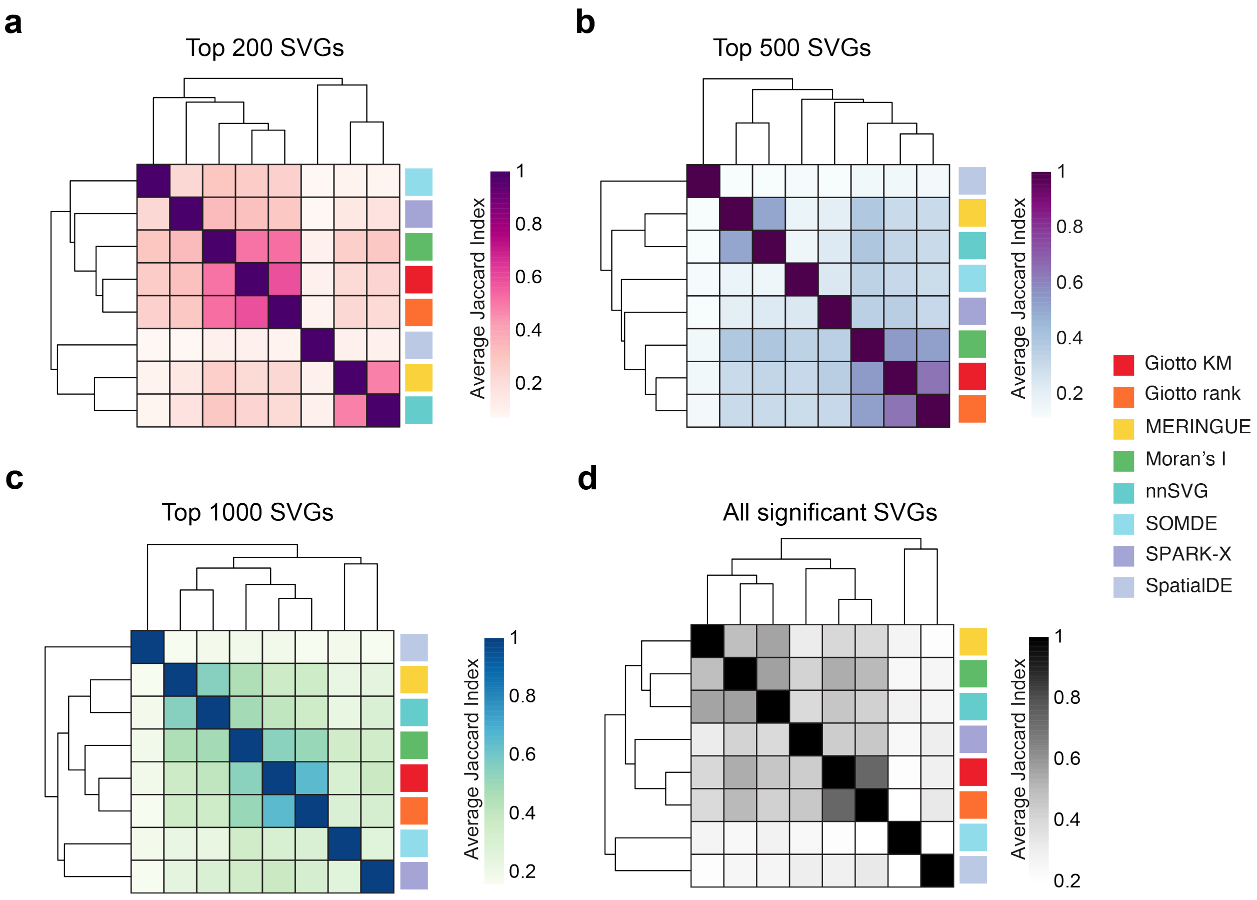

Fig. S5. Heatmaps of the overlap of SVGs reported by each method for each spatial transcriptomics dataset. The overlap calculated as the Jaccard Index was computed for the (**a**) top 200 SVGs, (**b**) top 500 SVGs, (**c**) top 1000 SVGs, and (**d**) all the significant SVGs (adjusted p-value <= 0.05) reported by each method for each spatial transcriptomics dataset. The average Jaccard Index across dataset and then plotted as a clustered hierarchical heatmap, where a higher Jaccard Index (darker colour) denotes a greater overlap of SVGs between methods and a lower Jaccard Index (lighter colour) denotes a lower overlap. The discrete colour bars denote the different SVG methods.

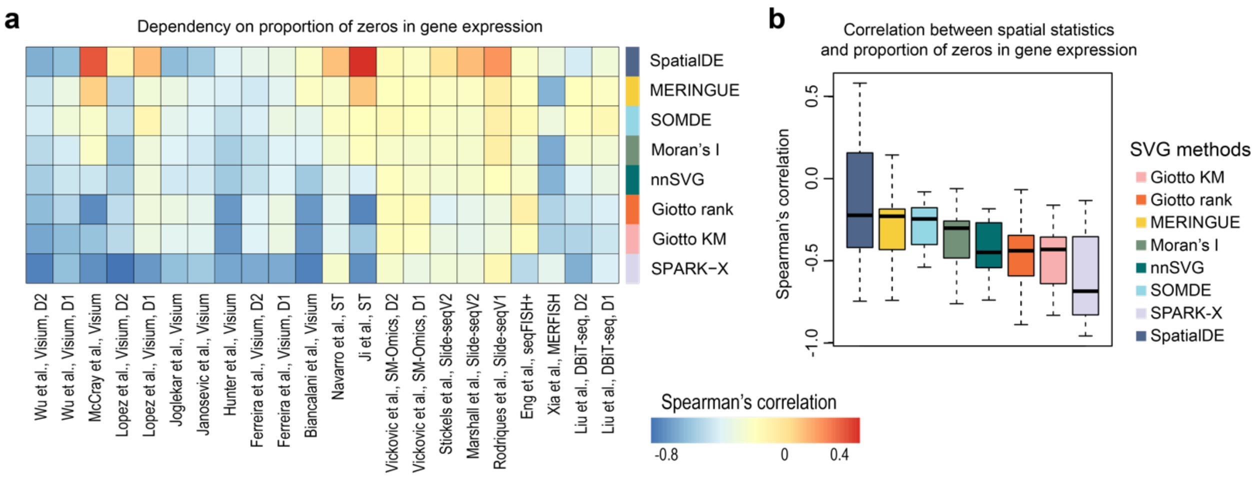

Fig. S6. Relationship between SVG statistics and proportion of zero of genes. (**a**) Heatmap summarising the Spearman's correlation of SVG statistics reported by each method and the proportion of zero of genes across cells in each dataset. (**b**) Boxplot of Spearman's correlation of SVG statistics reported by each method and the proportion of zero of genes across the spatial transcriptomics datasets.

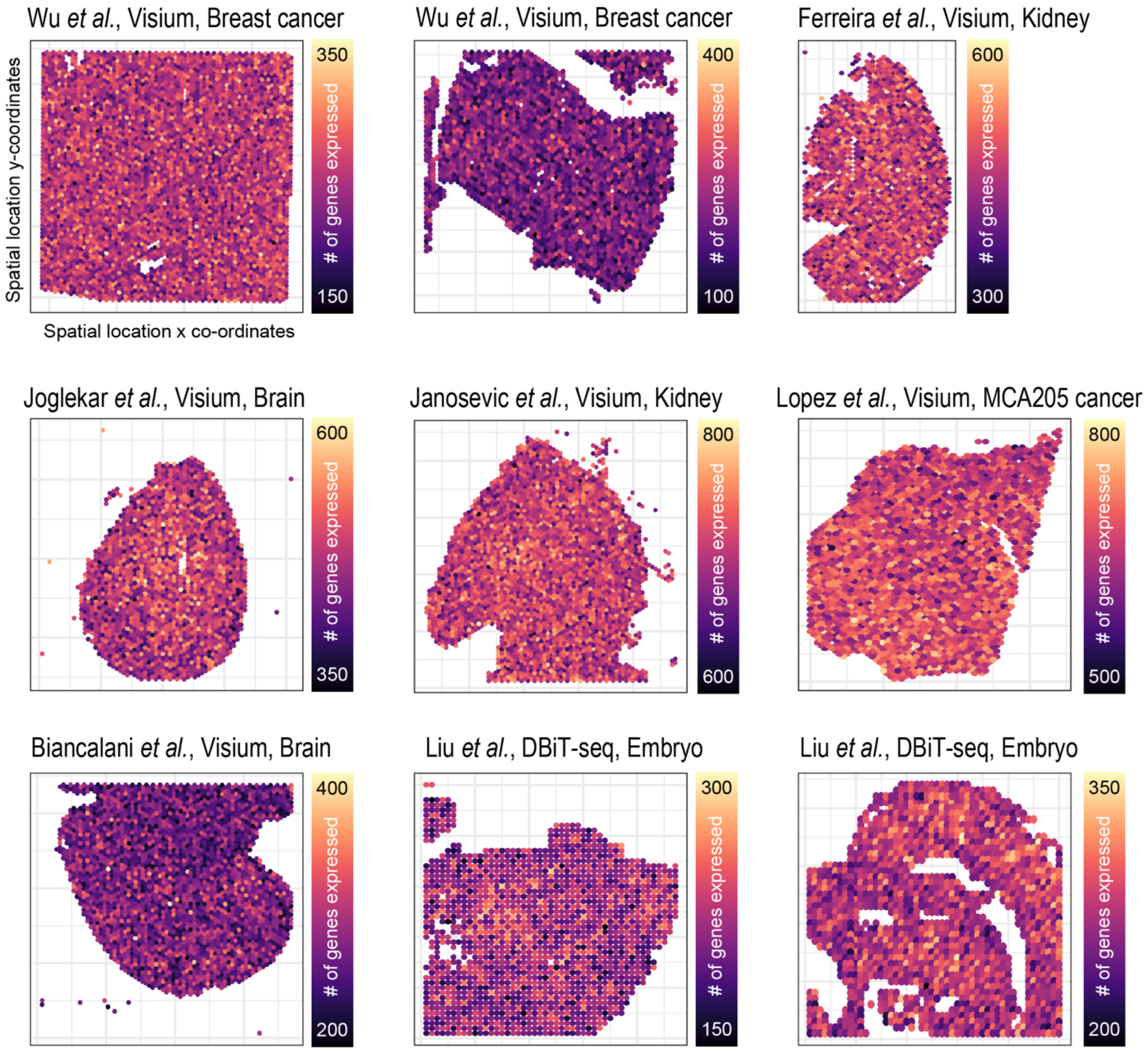

Fig. S7. Simulation of spatial transcriptomics data. Spatial locations and the total number of genes expressed per spatial spot in 9 simulated spatial transcriptomics datasets generated from different sources of data.

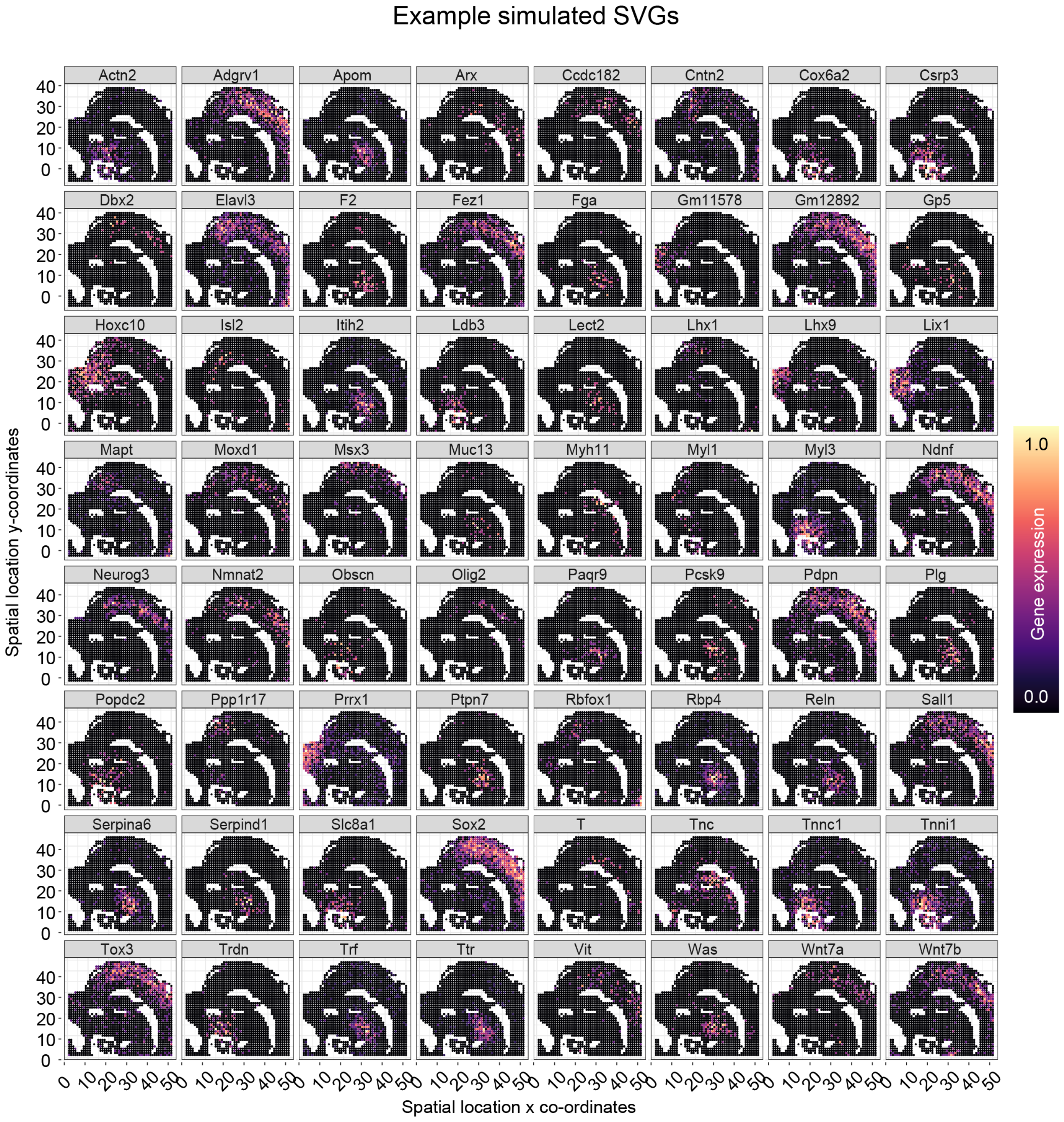

Fig. S8. Spatial patterns of spatially variant genes. Spatial expression patterns of example spatially variable genes of an example simulated spatial transcriptomics data (derived from Liu et al., DBiT-seq, Embryo).

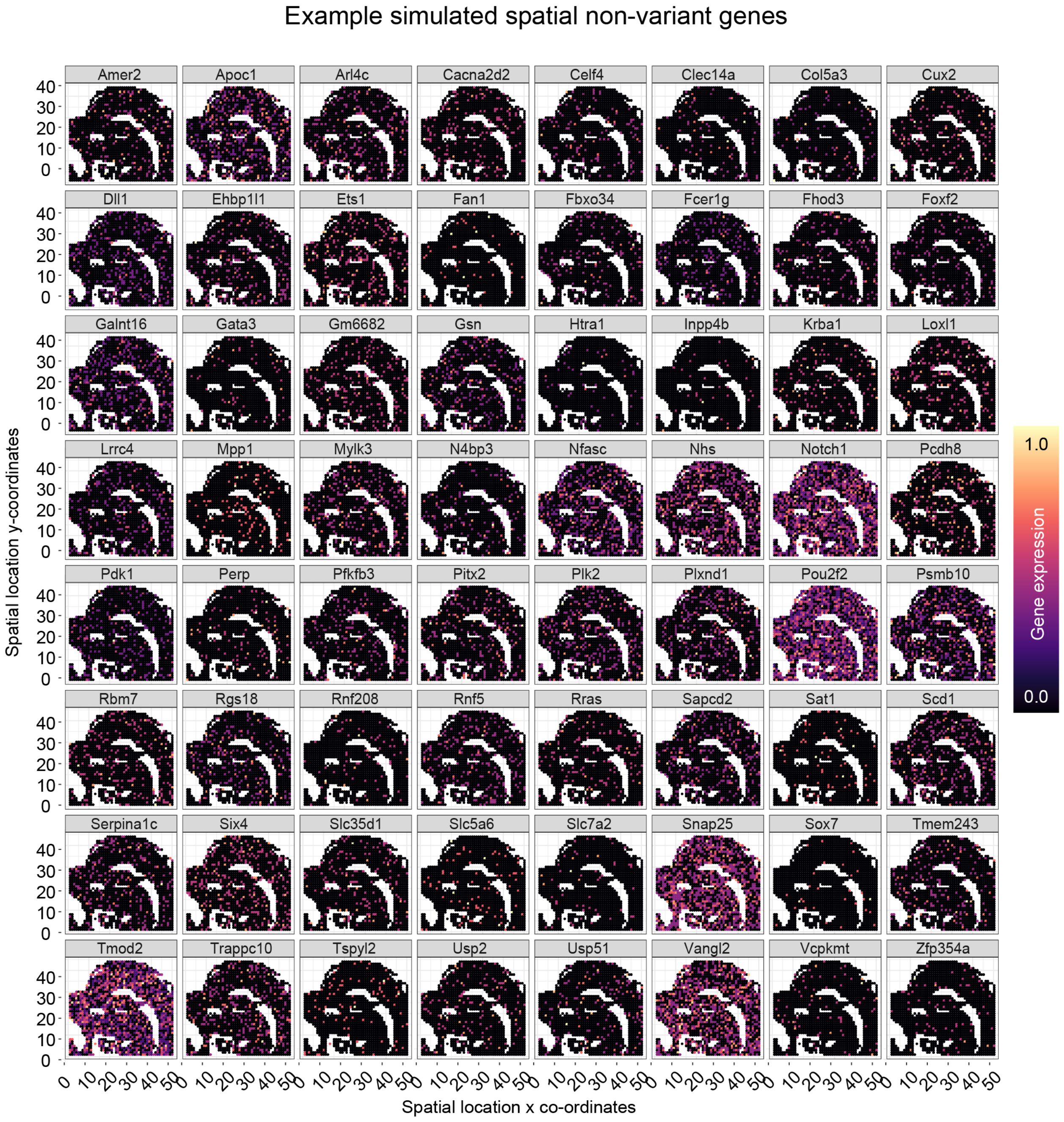

Fig. S9. Spatial patterns of spatially invariant genes. Spatial expression patterns of example non-spatially variable genes of an example simulated spatial transcriptomics data (derived from Liu et al., DBiT-seq, Embryo).

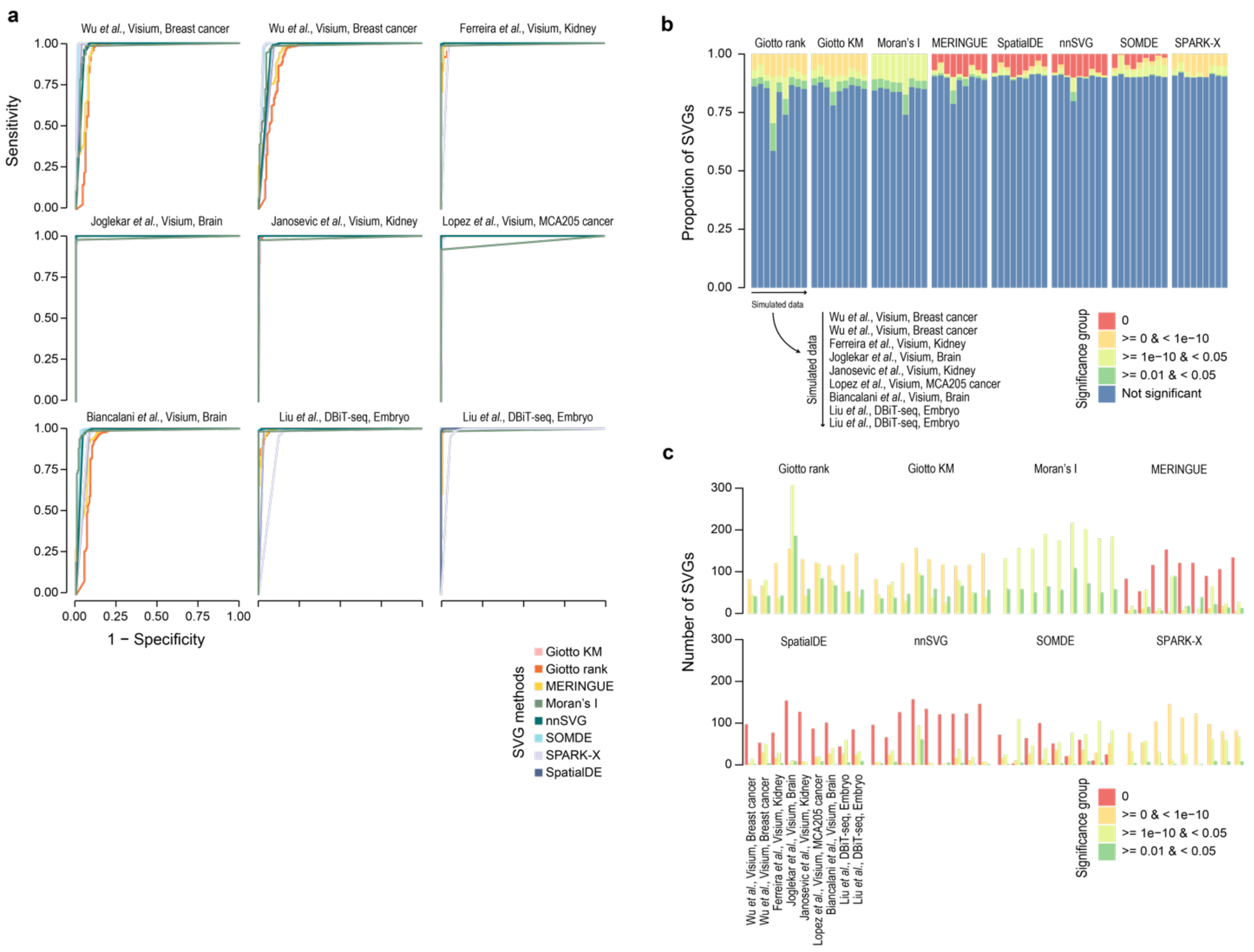

Fig. S10. ROC curves of spatially variable gene detection. (**a**) ROC curves were used to assess the capacity of each method to detect spatially variable genes between two spatial domains. The curves are colour-coded by methods. The 1-specificity and sensitivity of spatially variable gene detection are plotted on the x- and y-axes, respectively. (**b**) Proportion bar plots of SVGs of different statistical significance levels identified by each method. SVGs are partitioned into five categories based on the adjusted p-values reported by each method (i.e., p = 0; 0 < p <= 1e-10; 1e-10 < p <= 0.01; 0.01 < p <= 0.05; and p > 0.05) and presented as a percentage (y-axis). (**c**) Bar plots of the total number of SVGs reported by each method, for each simulated data, and for each statistical significance level as in (**b**).

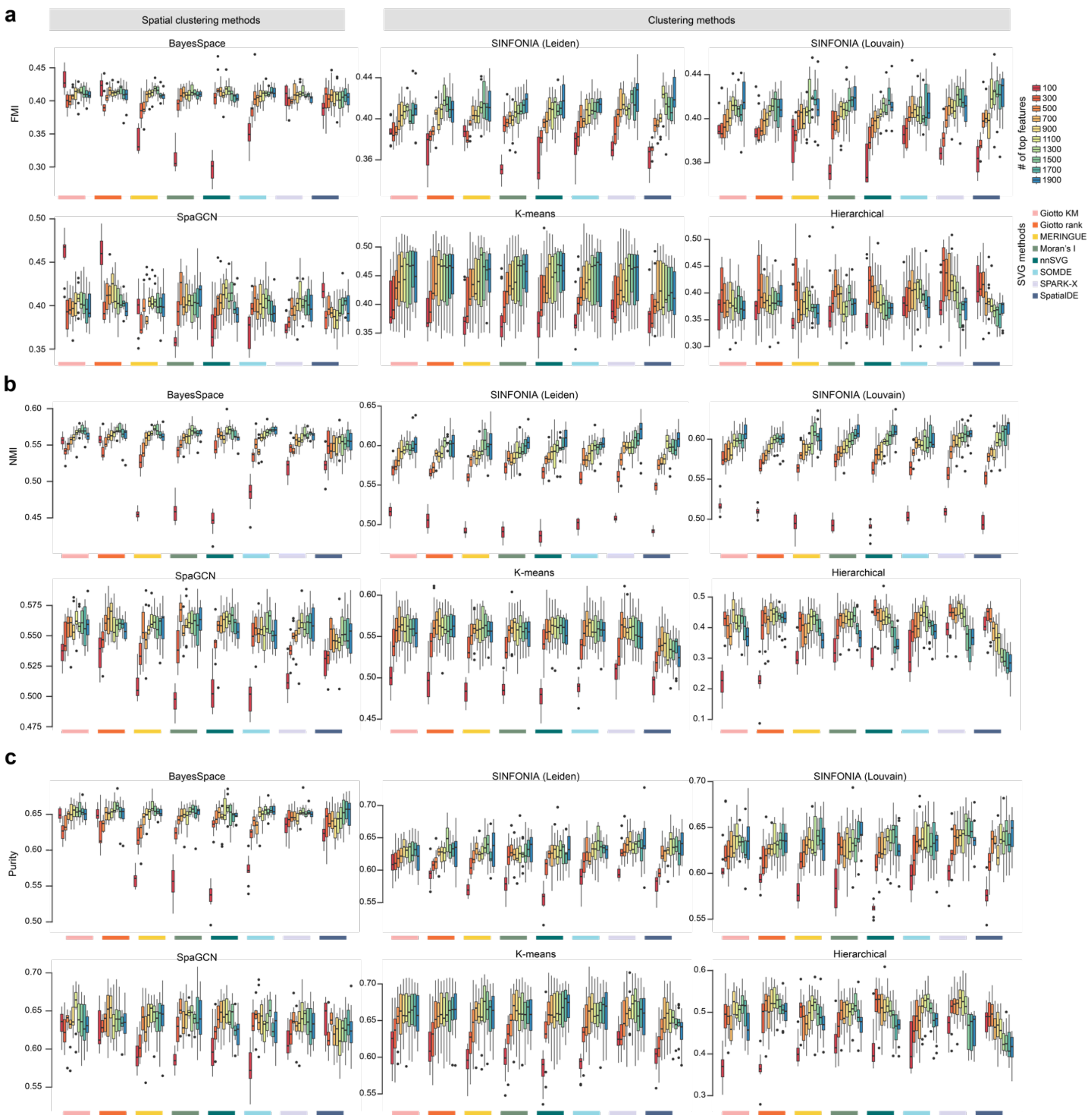

Fig. S11. Performance of SVGs selected by each SVG method for clustering spatial domains in the mouse embryo. Concordance in the clustering outputs from spatial clustering methods (BayesSpace and SpaGCN) and non-spatial clustering methods (SINFONIA's Louvain and Leiden, k-means, and hierarchical clustering) and the pre-defined spatial domains in the mouse embryo was computed using across a range of top SVGs (between 100 and 1900

genes). Concordance was quantified by three metrics including (**a**) Fowlkes-Mallows index (FMI), (**b**) normalized mutual information (NMI), and (**c**) purity.
