# Supplementary material for: Evaluating spatially variable gene detection methods for spatial transcriptomics data
Source: Genome Biol. 2024 Jan 15;25:18. doi: 10.1186/s13059-023-03145-y (PMC10789051; doi:10.1186/s13059-023-03145-y)
Supplement: Supplementary file 2 — Additional file 2. Review history. [file 13059_2023_3145_MOESM2_ESM.docx]

Review History

**First round of review**

**Reviewer 1**

**Were you able to assess all statistics in the manuscript, including the appropriateness of statistical tests used?**

Yes, and I have assessed the statistics in my report.

**Comments to author:**

In this review, the authors systematically evaluated seven spatially variable gene (SVG) detection methods on multiple simulated spatial transcriptomic (ST) datasets and real-world ST datasets of various tissues, sequencing technologies and spatial resolutions. They designed comprehensive and comparative experiments to validate these SVG detection methods on key problems including the concordance of SVGs detected by different methods, the dependency of SVG statistics on gene expression levels, the interdependency of genes across different methods, the accuracy in selecting ground-truth SVGs based on simulated datasets, the performance of detected SVGs on downstream clustering tasks, and the computational time and memory efficiency. Altogether, this is an impressive review providing useful information for understanding, choosing, and developing SVG detection methods. It would be better if the following issues can be addressed.

Major comments:

1. As described in lines 209-218, the authors stated that they directly implemented a clustering algorithm on detected SVGs (or top-k SVGs) to obtain spatial domains for the evaluation of SVG detection methods on downstream clustering. However, when analyzing both single-cell transcriptomics data and spatial transcriptomics data, dimension reduction algorithms, such as PCA, are usually performed between the SVG (or HVG) selection step and clustering step to obtain an improved clustering result [1-3]. Therefore, it would be more convincing, from my point of view, if a common dimension reduction method such as PCA is performed before clustering for the evaluation of SVG detection methods on clustering spatial domains [3].

2. As noted in lines 214-220, most SVG detection methods provide relatively poor clustering performance based on all significant SVGs, while selecting top-500 or top-100 SVGs led to improved results. Consistent with this conclusion, current widely-used tools for analyzing single-cell and spatial transcriptomics data also recommend choosing top-k SVGs (or HVGs) [1, 2]. Researchers may be interested in how many SVGs should be selected for downstream analyses. Therefore, more experiments and suggestions on the choice of number of SVGs would be helpful.

3. Compared with the k-means algorithm, the community-based clustering algorithms, i.e., Louvain or Leiden, are more commonly used to obtain cell clusters or spatial domains in the field of spatial and single-cell transcriptomics [1-4]. Hence, it would be more convincing if the evaluation of SVG detection methods on downstream clustering utilizes Louvain or Leiden algorithm besides k-means algorithm.

4. The authors utilized four clustering metrics, including ARI, FMI, NMI, and Purity, to systematically evaluate the performance of clustering results of different SVG detection methods. It would be better to explain how these scores are calculated in the manuscript, discuss the advantages or disadvantages of different metrics [5, 6], and provide the source of the scores for readers' reference.

5. As far as I know, SPARK [7] is known as an effective and popular SVG detection method to decipher spatial expression patterns and SINFONIA [3] is a recently developed ensemble method that integrates the Moran's I and Geary's C to identify SVGs on Python platform. Due to the effectiveness of the two methods and their close relation to this review, it would be better to add these two methods for further evaluation and comparison.

6. The authors should publicize all the scripts and provide documents for the reproduction of the benchmark.

Minor comments:

1. It would be better if there is a space as an interval between the quoted word and the corresponding quotation mark.

2. There are no letters in Figure 4 to indicate different panels including Figure 4a and Figure 4b.

3. Before using the abbreviation of a noun, it is better to state it completely in front to avoid ambiguity, such as adjusted rand index (ARI).

Reference:

[1] Wolf FA, Angerer P, Theis FJ: SCANPY: large-scale single-cell gene expression data analysis. Genome Biol 2018, 19:1-5.

[2] Hao Y, Hao S, Andersen-Nissen E, Mauck III WM, Zheng S, Butler A, Lee MJ, Wilk AJ, Darby C, Zager M: Integrated analysis of multimodal single-cell data. Cell 2021, 184:3573-3587. e3529.

[3] Jiang R, Li Z, Jia Y, Li S, Chen S: SINFONIA: Scalable Identification of Spatially Variable Genes for Deciphering Spatial Domains. Cells 2023, 12:604.

[4] Dong K, Zhang S: Deciphering spatial domains from spatially resolved transcriptomics with an adaptive graph attention auto-encoder. Nat Commun 2022, 13:1-12.

[5] Chen S, Yan G, Zhang W, Li J, Jiang R, Lin Z: RA3 is a reference-guided approach for epigenetic characterization of single cells. Nat Commun 2021, 12:2177.

[6] Chen S, Zhang B, Chen X, Zhang X, Jiang R: stPlus: reference-based enhancement of spatial transcriptomics. Bioinformatics 2021, 37(S1):i299-i307.

[7] Sun S, Zhu J, Zhou X: Statistical analysis of spatial expression patterns for spatially resolved transcriptomic studies. Nat Methods 2020, 17:193-200.

**Reviewer 2**

**Were you able to assess all statistics in the manuscript, including the appropriateness of statistical tests used?**

Yes, and I have assessed the statistics in my report.

**Comments to author:**

Chen et al. presents a benchmark study to compare methods for detecting spatially variable genes. This is an important topic, but there is much room for improvement in the depth of the analyses presented. The current real data analyses focus on the concordance between different methods, but without ground truth information, it's hard to think how potential users can select methods based on the presented results. The simulation study is based on a single dataset, and therefore, also provides limited insights for real practice. My detailed comments are listed below.

The manuscripts claim that 20 spatial transcriptomics datasets were used for evaluation, but 6 of them were different mouse samples from the same study. Counting them as 6 separate datasets may bias the conclusions. A related question is that the majority of the 20 datasets were from the Visium technology, so methods perform well on this technology will have an advantage in this study. It would be more useful to compare methods based on a more balanced dataset in terms of both technologies and data generation teams.

It would be helpful to include other methods such as SPARK and MERINGUE in the analysis to provide a more comprehensive comparison.

"we first obtained the ranking of genes in each dataset … based on the statistics reported by each method": What are the statistics used to rank genes? Are they p values or other statistics? Are these the same type of statistics calculated by different methods? A detailed description should be provided in Methods.

The manuscript claims that "datasets generated using the Visium platform result in a more consistent SVG ranking". This argument may not be reliable as (1) These datasets have different number of genes, and (2) These datasets may have different sequencing depths, which would influence methods performance.

Related to the comment above, it would be helpful to see methods concordance based on the top 200/500/1000 genes, as many potential users of these methods would focus on the top genes in downstream analysis instead of the complete ranked list.

The authors used adjusted p values reported by each method. As the methods may have used different approaches for p value adjustment, it would be helpful to redo the adjustment using the same approach, so that the results are not confounded by difference in p value adjustment.

The downsampling experiment could provide useful information to potential users, but it was only performed on a single dataset. A more comprehensive evaluation is needed. In addition, the number of genes that can be sequenced in a technology typically does not change much, so it would be more interesting to downsample cells/spots instead of genes.

The simulation study includes the single analysis that provides a chance to evaluate the accuracy of SVG methods. However, it was again based on a single dataset and did not reflect the diversity of sequencing technologies and tissue histology. The number of genes, which was set to 500, also did not reflect the actual gene number in real data.

**Authors Response**

**Point-by-point responses to the reviewers’ comments:**

Reviewer #1:
In this review, the authors systematically evaluated seven spatially variable gene (SVG) detection methods on multiple simulated spatial transcriptomic (ST) datasets and real-world ST datasets of various tissues, sequencing technologies and spatial resolutions. They designed comprehensive and comparative experiments to validate these SVG detection methods on key problems including the concordance of SVGs detected by different methods, the dependency of SVG statistics on gene expression levels, the interdependency of genes across different methods, the accuracy in selecting ground-truth SVGs based on simulated datasets, the performance of detected SVGs on downstream clustering tasks, and the computational time and memory efficiency. Altogether, this is an impressive review providing useful information for understanding, choosing, and developing SVG detection methods. It would be better if the following issues can be addressed.

Response: We thank the reviewers for their positive view towards our manuscript. We have taken onboard all suggested points and responded point-by-point below.

Major comments:

1. As described in lines 209-218, the authors stated that they directly implemented a clustering algorithm on detected SVGs (or top-k SVGs) to obtain spatial domains for the evaluation of SVG detection methods on downstream clustering. However, when analyzing both single-cell transcriptomics data and spatial transcriptomics data, dimension reduction algorithms, such as PCA, are usually performed between the SVG (or HVG) selection step and clustering step to obtain an improved clustering result [1-3]. Therefore, it would be more convincing, from my point of view, if a common dimension reduction method such as PCA is performed before clustering for the evaluation of SVG detection methods on clustering spatial domains [3].

Response: We thank the reviewer for this comment. As suggested, we have now performed a uniform dimension reduction step, using principal component analysis, for the datasets after selecting the SVGs and then performed clustering on the 20 principal components. We note that generally the clustering results have improved across SVG detection methods and metrics. We have replaced the old results with these new updates in Figure 6 of the manuscript (please see Response Point 2 for the corresponding figure) and have now updated the description of the clustering approach in the Methods section.

2. As noted in lines 214-220, most SVG detection methods provide relatively poor clustering performance based on all significant SVGs, while selecting top-500 or top-100 SVGs led to improved results. Consistent with this conclusion, current widely-used tools for analyzing single-cell and spatial transcriptomics data also recommend choosing top-k SVGs (or HVGs) [1, 2]. Researchers may be interested in how many SVGs should be selected for downstream analyses. Therefore, more experiments and suggestions on the choice of number of SVGs would be helpful.

Response: To provide a recommendation on the most optimal number of SVGs that should be selected for downstream analyses, we performed a systematic evaluation of the impact of the number of SVGs on the clustering of pre-defined spatial domains of the mouse embryo. By incrementally increasing the number of top SVGs used from 100 to 2,000, we demonstrate that the most optimal number of SVGs is approximately between 900 and 1300 genes across all methods. The accuracy of clustering did not fall below an adjusted rand index of 0.35, an NMI of 0.55, an FMI of 0.45, and a purity score of 0.65 after 1100-1300 SVGs for most methods except SpatialDE. These findings suggest that the optimal number of SVGs is largely consistent between SVG methods. Most methods performed comparably to each other across the gene range investigated.

This analysis was repeated with hierarchical clustering in place of k-means clustering (Supplementary Figure 10). We demonstrate the optimal number of SVGs is largely consistent when using hierarchical clustering, although the range in SVG peak performance was more variable between methods (approximately 300-1100 SVGs). We have now edited the manuscript to describe these new findings (Figure 6 and Supplementary Figure 10) and to make a recommendation for the number of SVGs for clustering analysis.

3. Compared with the k-means algorithm, the community-based clustering algorithms, i.e., Louvain or Leiden, are more commonly used to obtain cell clusters or spatial domains in the field of spatial and single-cell transcriptomics [1-4]. Hence, it would be more convincing if the evaluation of SVG detection methods on downstream clustering utilizes Louvain or Leiden algorithm besides k-means algorithm.

Response: We acknowledge that Louvian and Leiden clustering algorithms are commonly used in analyzing spatial and single-cell transcriptomics data. However, the inability to pre-define the number of clusters in community detection methods means that the number of final clusters will vary across results, making it hard to interpret the clustering performance on selected SVGs (i.e., different number of clusters lead to highly variable clustering performance). For example, when the resolution is set to 0.8, the total number of clusters detected by the Louvain clustering varies between 4 and 13 clusters, depending on the SVG selection methods, number of SVGs, and replicates. With 15 replicates and 10 varying features selected by 8 different SVG detection methods, it would be extremely challenging to evaluate and interpret the impact/utility of SVGs using Louvian and Leiden clustering algorithms. Since the goal of the evaluation is to compare between SVG evaluation methods and not clustering methods, the choice of k-means clustering, which allows the number of clusters to be pre-defined (in our case defined as the number of spatial domains in a dataset), alleviates the difficulties associated with the comparison of the accuracies between methods and metrics when results contain different numbers of clusters. To further validate these results, we have now also performed hierarchical clustering analysis using selected SVGs and split the clustering results to the same number of clusters for quantifying their performance. These results are now included in Figure 6 (k-means clustering) and Supplementary Figure 10 (hierarchical clustering).

4. The authors utilized four clustering metrics, including ARI, FMI, NMI, and Purity, to systematically evaluate the performance of clustering results of different SVG detection methods. It would be better to explain how these scores are calculated in the manuscript, discuss the advantages or disadvantages of different metrics [5, 6], and provide the source of the scores for readers' reference.

Response: We have revised the Methods to include a more detailed description of how these metrics (ARI, FMI, NMI, and purity) work, referenced the relevant sources, and provided a brief discussion of the advantages and disadvantages of these metrics for the readers’ reference. Please see Methods subsection “Clustering and concordance quantification”.

5. As far as I know, SPARK [7] is known as an effective and popular SVG detection method to decipher spatial expression patterns and SINFONIA [3] is a recently developed ensemble method that integrates the Moran's I and Geary's C to identify SVGs on Python platform. Due to the effectiveness of the two methods and their close relation to this review, it would be better to add these two methods for further evaluation and comparison.

Response: We thank the reviewer for their suggestions to improve the comprehensiveness of our benchmarking paper. We have considered SINFONIA as possible SVG methods to benchmark. SINFONIA is an ensemble method that computes Moran’s I and Geary’s C score and then takes the union of the top ‘n’ SVGs (where ‘n’ is a user defined value) identified by the two approaches. In our benchmarking analysis, SINFONIA was excluded for several reasons: 1) the method does not report P values; 2) the method does not report a rank or test statistics; 3) the method reports only a set of spatially variable genes; and 4) the method requires the user to arbitrarily select a top number of SVGs to detect. Importantly, the lack of P values or an approach to rank the spatial variable genes precludes SINFONIA from all the benchmarking evaluations except the computational time benchmarking. Therefore, SINFONIA was excluded from our study.

SPARK-X (PMID: 34154649), an update of SPARK proposed by the same group of authors, was included in our benchmark study. A key development in recent SVG methods is computational scalability, which is becoming more relevant with the increased commercialisation of latest spatial resolution technologies (e.g., 10x Genomics Visium and Slide-seqV2) containing thousands of spatial locations per tissue. As noted by the original authors, “SPARK scales cubically with the number of spatial locations” (PMID: 34154649), whilst their newer implementation, SPARK-X, scales linearly. As such, SPARK-X is significantly faster than SPARK; the original authors show that SPARK-X is ~1500 times faster than SPARK-X (see Figure 3). They further showed that SPARK-X outperforms SPARK to detect various spatial patterns and spatial expression genes in larger datasets, whilst demonstrating comparable power to detect hotspot patterns in smaller datasets (PMID: 34154649). For these reasons, we have chosen to benchmark its update SPARK-X.

In addition, we have now also included MERINGUE (PMID: 3403504), another recent and popular SVG detection method. Briefly, we demonstrate that MERINGUE statistics is overall most similar to nnSVG and Moran’s I (Figure 2 and Supplementary Figure 4). MERINGUE was one of the methods whose spatial statistics were least correlated to the mean gene expression (Figure 3), and it was among the most reproducible methods with induced downsampling of genes and demonstrated intermediate robustness against induced downsampling of spatial spots (Figure 4). In terms of identifying the simulated SVGs, whilst MERINGUE was able to identify the vast majority of significant SVGs, it did so with a high false discovery rate, identifying a high number of false positives and ranked fourth in terms of the false discovery rate, which distinguished the high and low performant methods (Figure 5). In terms of identifying SVGs that can accurately cluster spatial domains, MERINGUE performed comparably to all other methods, except SpatialDE (Figure 6). In terms of memory and computational speed, MERINGUE ranked 6th, being one of the slow and memory intensive methods (Figure 7). Interpretation of the results for MERINGUE have been incorporated into all the relevant Results sections of the revised manuscript.

6. The authors should publicize all the scripts and provide documents for the reproduction of the benchmark.

Response: We have now provided reproducible code in our Github repository (https://github.com/PYangLab/SVGbench) that can be used to reproduce figures presented in our paper.

Minor comments:
1. It would be better if there is a space as an interval between the quoted word and the corresponding quotation mark.

Response: We thank this suggestion and will consult the editor for the journal formatting requirement.

2. There are no letters in Figure 4 to indicate different panels including Figure 4a and Figure 4b.

Response: We have added the panel labels in Figure 5 (the new Figure 4) and updated the legend accordingly.

3. Before using the abbreviation of a noun, it is better to state it completely in front to avoid ambiguity, such as adjusted rand index (ARI).

Response: We have expanded all abbreviations at their first instance.

Reviewer #2:
Chen et al. presents a benchmark study to compare methods for detecting spatially variable genes. This is an important topic, but there is much room for improvement in the depth of the analyses presented. The current real data analyses focus on the concordance between different methods, but without ground truth information, it's hard to think how potential users can select methods based on the presented results. The simulation study is based on a single dataset, and therefore, also provides limited insights for real practice. My detailed comments are listed below.

Response: We thank the reviewers for the constructive comments. We have taken on all suggestions from the reviewer as detailed in the point-by-point response below and performed additional experiments (e.g. expanding the simulations) and revised the manuscript according to these comments.

1. The manuscripts claim that 20 spatial transcriptomics datasets were used for evaluation, but 6 of them were different mouse samples from the same study. Counting them as 6 separate datasets may bias the conclusions. A related question is that the majority of the 20 datasets were from the Visium technology, so methods perform well on this technology will have an advantage in this study. It would be more useful to compare methods based on a more balanced dataset in terms of both technologies and data generation teams.

Response: We thank the reviewer for this comment. To reduce the bias from tissue types, we have now removed 4 out of 6 samples from the Wu et al. study that were all from mouse breast cancer tissue. Furthermore, we have now expanded the number of datasets generated from other spatial platforms, including Slide-seq (PMID:30923225) Slide-seqV2 (PMID:35372810), DBiT-seq (PMID:33188776), and SM-Omics (PMID:35145087), to alleviate the platform imbalance among the spatial datasets tested. Nevertheless, we note that the Visium platform is the most widely commercialized spatial technology as is reflected by the number of datasets generated by this platform. The final benchmarking datasets comprise of eight platform types including MERFISH, seqFISH+, Slide-seqV1, Slide-seqV2, Visium, Spatial Transcriptomics (ST), DBiT-seq, and scOmics, and a large number of tissue types including the embryo, brain, kidney, prostate, lymph node, and various cancer tissues across three species (zebrafish, mouse, and human). The revised Supplementary Figure 1 summarizes the datasets used and the proportion of platform and tissue types covered in the benchmarking study.

2. "we first obtained the ranking of genes in each dataset … based on the statistics reported by each method": What are the statistics used to rank genes? Are they p values or other statistics? Are these the same type of statistics calculated by different methods? A detailed description should be provided in Methods.

Response: We have now included Table 1 in the Method section describing the statistics and p-values generated by each method. We hope that the table provides clarity in the way that the methods were compared. Please see Methods subsection “SVG detection methods”.

3. The manuscript claims that "datasets generated using the Visium platform result in a more consistent SVG ranking". This argument may not be reliable as (1) These datasets have different number of genes, and (2) These datasets may have different sequencing depths, which would influence methods performance.

Response: We thank the reviewer for pointing this out. We have now removed this claim from our manuscript.

4. Related to the comment above, it would be helpful to see methods concordance based on the top 200/500/1000 genes, as many potential users of these methods would focus on the top genes in downstream analysis instead of the complete ranked list.

Response: We thank the reviewer for this comment. We have now highlighted the concordance based on the top 200, 500, 1000 genes as well as all SVGs identified as significant by each SVG method. As we have included an additional method (MERINGUE) in the revision, we have now adopted a heatmap of pairwise Jaccard indices (of the average across all datasets) instead of a Venn diagram for ease of visualization (Supplementary Figure 4). The Jaccard index, or the Jaccard similarity coefficient, is a statistical measure used to compare the similarity between the two sets. As the Jaccard Index takes into account the relative sizes of the sets by considering the ratio of the intersection to the union, the scale-independence makes it a more reliable measure of overlap than the absolute number of overlap.

Our findings show that irrespective of the top number of SVGs used, the overall pattern in similarity between methods remains consistent. We found that SpatialDE generated gene sets that were most unique compared to other methods, branching out earliest in the hierarchical tree. This finding is consistent with our observation in Figure 2A, where spatialDE demonstrated the lowest overall correlation against the other methods. We observed two categories of methods that generated similar SVG sets, 1) MERINGUE and nnSVG; and 2) Giotto KM, Giotto Rank, and Moran’s I. Intriguingly, Moran’s I, which showed a highest correlation with nnSVG in our correlation result (across all genes), showed a stronger concordance with the Giotto methods when measuring the overlap in the top gene sets (especially for top 200 and 500 SVGs). A greater concordance with nnSVG than the Giotto methods is observed when all significant SVGs are compared, suggesting that whilst the overall ranking in gene statistics may be similar between nnSVG and Moran’s I, the top most significant SVGs identified by Moran’s I appear to be more similar to those of the Giotto methods. Finally, SOMDE and SPARK-X demonstrated moderate overlap against the other methods. These results are now incorporated in the revised manuscript.

5. It would be helpful to include other methods such as SPARK and MERINGUE in the analysis to provide a more comprehensive comparison.

Response: We thank the reviewer for this suggestion. We have included SPARK-X in our benchmark study, which is the update of SPARK by the same group of authors. As noted by the authors, “SPARK scales cubically with respect to the number of spatial locations” (PMID: 34154649) and is becoming increasingly less scalable to datasets generated from 10x Genomics Visium and Slide-seqV2 that contain thousands of spatial locations per tissue. This has motivated them to develop a more efficient version called SPARK-X that scales linearly. In particular, SPARK is significantly slower than SPARK-X; the original authors show that 1) SPARK-X is ~1500 times faster than SPARK; 2) SPARK-X outperforms SPARK to detect various spatial patterns and spatial expression genes in larger datasets; and 3) SPARK-X and SPARK demonstrate “comparable power” to detect hotspot patterns in smaller datasets (PMID: 34154649). Given the computational efficiency and superiority capacity of SPARK-X to determine spatial genes, we believe the inclusion of SPARK-X is more relevant to our benchmark study.
As suggested, we have now also included MERINGUE (PMID: 3403504), another recent and popular SVG detection method. Briefly, we demonstrate that MERINGUE statistics is overall most similar to nnSVG and Moran’s I (Figure 2 and Supplementary Figure 4). MERINGUE was one of the methods whose spatial statistics were least correlated to the mean gene expression (Figure 3), and it was among the most reproducible methods with induced downsampling of genes and demonstrated intermediate robustness against induced downsampling of spatial spots (Figure 4). In terms of identifying the simulated SVGs, whilst MERINGUE was able to identify the vast majority of significant SVGs, it did so with a high false discovery rate, identifying a high number of false positives and ranked fourth in terms of the false discovery rate, which distinguished the high and low performant methods (Figure 5). In terms of identifying SVGs that can accurately cluster spatial domains, MERINGUE performed comparably to all other methods, except SpatialDE (Figure 6). In terms of memory and computational speed, MERINGUE ranked 6th, being one of the slow and memory intensive methods (Figure 7). Interpretation of the results for MERINGUE have been incorporated into all the relevant Results sections of the revised manuscript.

6. The authors used adjusted p values reported by each method. As the methods may have used different approaches for p value adjustment, it would be helpful to redo the adjustment using the same approach, so that the results are not confounded by difference in p value adjustment.

Response: We thank the reviewer for this comment. It is true that the SVG detection methods may report p-values using different multiple testing correction methods. However, we would like to stress that the way in which the SVGs are detected by each method is vastly different, meaning the approach to p-value adjustment is not the only confounder in the results. Further, we believe that the methods should be benchmarked using the p-value adjustment selected by the original authors, which is also the approach used to test the rigor of the methods in their respective research articles. Therefore, we have chosen to use the default settings in each method for multiple testing corrections.

7. The downsampling experiment could provide useful information to potential users, but it was only performed on a single dataset. A more comprehensive evaluation is needed. In addition, the number of genes that can be sequenced in a technology typically does not change much, so it would be more interesting to downsample cells/spots instead of genes.

Response: We agree that the downsampling of cells/spots would provide interesting insight into the robustness of the SVG detection methods against the sparsity of spatial spots captured by the spatial sequencing technologies. Whilst dropouts in spatial spots is uncommon, inefficiencies in sample preparation may lead to some spots being compromised. Therefore, to simulate such a scenario, we performed downsampling of cells/spots to 80% of the total number of cells/spots and then repeated the SVG calling.

We visualized the results at two levels: 1) the correlation of overall SVG statistics and 2) the proportion of significant SVGs determined from the subsampling data that are either present or absent in the full data. Our results show that even with 20% of the spatial spots removed all of the methods were fairly robust against the induced sparsity, showing greater than 0.8 correlation in spatial statistics for the Wu et al. dataset (Figure 4e-f). Using another dataset (Stickels et al.), the results were more pronounced with all methods affected by the induced sparsity. The methods that were less sensitive were SPARK-X, SpatialDE, and Moran’s I, whilst methods that rely on neighborhood adjacency relationships like nnSVG, SOMDE, MERINGUE, and the Giotto methods were more sensitive. nnSVG uses spatial covariance functions in Gaussian Processes using a nearest-neighbor Gaussian process (NNGP) model, SOMDE uses self-organizing map to cluster neighboring cells into nodes, MERINGUE uses neighborhood relationships using Voronoi tessellation, and the Giotto methods rely on creating a Delaunay triangulation network, representing a spatial network constructed based on cell centroid physical distances. The reliance on nearest neighbors or distance-based networks may explain the sensitivity to sparsity as it affects the detection of SVGs based on its expression between neighbors in a spatial network. We further investigated the capacity of these methods to avoid the detection of false positives by calculating the number of spatial genes identified that are not identified in the full spatial data. We found that SPARK-X and SpatialDE performed the best, identifying very few false positives in both datasets.

For both the spot-wise and gene-wise downsampling experiments, we have performed the analysis in two spatial transcriptomics data derived from differing technologies. These findings have now been incorporated into Figure 4 in the manuscript and described in the Results section. The full description of the experiments can be found in the Methods under the sections “Dependency across genes” and “Robustness against sparsity”.

8. The simulation study includes the single analysis that provides a chance to evaluate the accuracy of SVG methods. However, it was again based on a single dataset and did not reflect the diversity of sequencing technologies and tissue histology. The number of genes, which was set to 500, also did not reflect the actual gene number in real data.

Response: We thank the reviewer for this comment. We have now significantly expanded our simulation study by including nine datasets from seven independent studies that cover different sequencing technologies (Visium and DBiT-seq) and tissue histologies (breast cancer, brain, embryo, and cancer). Furthermore, we have now implemented the recently published scDesign3 framework (PMID: 37169966), a simulator of spatial transcriptomics data, to generate the simulation datasets. The scDesign3 simulator enables generation of diverse and realistic spatial transcriptomics data. Our simulation datasets now cover 590-1937 genes, 59-194 spatially variable genes, 369-4895 spatial spots, and diverse spatial structures (Figure 5, Supplementary Figure 6-9). Our revised simulation framework now includes simulated data that reflects the diversity of spatial transcriptomics data and technologies.

Consistent with our previous findings, our new simulation results (Figure 5a) show that SPARK-X and SOMDE are among the top performing methods, both identifying simulated SVGs with high true positive rate (TPR) and low false discovery rate (FDR). Whilst SpatialDE and nnSVG performed well on identifying the correct SVGs, both these methods demonstrated less optimal FDR control on some datasets. Despite demonstrating a high TPR, we again saw that Giotto methods and Moran’s I demonstrated poor control for the false discovery rate for every dataset. At FDR = 0.05, approximately 30% of SVGs identified as significant were false positives (Figure 5b). Whilst MERINGUE performed better than these methods but still did poorly in terms of FDR control. We have revised the manuscript (Results and Methods) to describe these new findings in Figure 5 and Supplementary Figures 6-9 in the manuscript.

**Second round of review**

**Reviewer 1**

I appreciate that the authors have thoroughly revised the manuscript based on the raised comments. Although the authors have additionally performed hierarchical clustering analysis using selected SVGs, I still suggest investigating the performance with community-based clustering algorithms, i.e., Louvain or Leiden, as they are more commonly used to obtain cell clusters or spatial domains in the field of spatial and single-cell transcriptomics. I agree that employing specific resolutions for clustering may pose challenges in evaluation. However, the authors could consider adopting a binary search method for tuning clustering resolution, similar to that utilized by SINFONIA, to obtain specific clusters in Louvain or Leiden clustering.

**Reviewer 2**

The revision and authors’ response have addressed some of my previous questions, but there are still two concerns that I suggest to be addressed to improve the quality of this work.

The revised manuscript has clarified about the method statistics used to calculate the concordance between different methods, but the current approach is not appropriate. Some of the statistics are based on p values adjusted by different multiple-testing correction methods, while others are observed/LLR statistics which are very different from p values. I don’t see how the spearman correlations between these statistics should be interpreted. To compare method in a fair way, it would make more sense to calculate correlation between raw p values or p values adjusted by the same multiple-testing correction method.

The manuscript extended the downsampling experiment from one dataset to two datasets, but we don’t know if the conclusions are generalizable. It would provide readers with a comprehensive comparison to perform the analysis across all datasets used in the study and summarize the results.

**Authors Response**

**Point-by-point responses to the reviewers’ comments:**

**Reviewer #1:**

1. I appreciate that the authors have thoroughly revised the manuscript based on the raised comments. Although the authors have additionally performed hierarchical clustering analysis using selected SVGs, I still suggest investigating the performance with community-based clustering algorithms, i.e., Louvain or Leiden, as they are more commonly used to obtain cell clusters or spatial domains in the field of spatial and single-cell transcriptomics. I agree that employing specific resolutions for clustering may pose challenges in evaluation. However, the authors could consider adopting a binary search method for tuning clustering resolution, similar to that utilized by SINFONIA, to obtain specific clusters in Louvain or Leiden clustering.

***Response:*** *We thank the reviewer for this suggestion. Using SINFONIA’s binary search method, we have performed Louvain and Leiden clustering and tuned the clustering resolution to match the number of spatial domains in the data. In addition to running these community base methods, we included two other spatially aware clustering methods (BayesSpace and SpaGCN) on the selected SVGs. We believe these results significantly enrich the clustering results using SVGs and demonstrate that whilst the optimal number of SVGs can vary between clustering methods and SVG selection tools, generally the optimal number of SVG is approximately 1000 SVGs. A new Figure 6 (also reproduced below) is now added to the manuscript and the texts are updated to incorporate these results (see section “Performance on clustering spatial domains”).*

**Figure 6. Performance of SVGs selected by each method for clustering spatial domains in mouse embryos.** **(a)** Proportion spatial locations annotated to one of sixteen tissue domains in the E9.5 mouse embryo. **(b)** Concordance measured in terms of the adjusted Rand index in the clustering outputs and the pre-defined spatial domains in the mouse embryo was computed across a range of top SVGs (between 100 and 1900 genes) selected by each method. Concordance of clustering outputs from six clustering methods and the pre-defined spatial domains are quantified.

**Reviewer #2:**

The revision and authors’ response have addressed some of my previous questions, but there are still two concerns that I suggest to be addressed to improve the quality of this work.

1. The revised manuscript has clarified about the method statistics used to calculate the concordance between different methods, but the current approach is not appropriate. Some of the statistics are based on p values adjusted by different multiple-testing correction methods, while others are observed/LLR statistics which are very different from p values. I don’t see how the spearman correlations between these statistics should be interpreted. To compare method in a fair way, it would make more sense to calculate correlation between raw p values or p values adjusted by the same multiple-testing correction method.

***Response:*** *As suggested, we have used the -log10 transformed raw p-values for all methods to calculate the Spearman’s correlation and plotted a side-by-side comparison of the pairwise boxplots comparing to the original results in our manuscript (Response Fig. 1). We note that in all cases except for Moran’s I (highlighted), the use of raw p-value does not change our analysis as the Spearman correlation considers the ranking of the genes, of which there is negligible difference regardless of whether the test statistic, adjusted p-values or raw p-values are used as is illustrated by additional examples (Response Fig. 2). We see that as we use raw p-values, Moran’s I exhibits poorer concordance with other methods, in particular MERINGUE and nnSVG. There is also lower reproducibility of the concordance across datasets.*

*However, we find it is not suitable to use the raw p-values for Moran’s I as the relationship between the p-values and observed coefficient is not directly proportional. As illustrated in Response Fig. 3A, the observed coefficient is a measure of spatial autocorrelation from a range of -1 to 1, with -1 representing significant negative spatial autocorrelation (even distribution of spatial variance), 0 representing insignificant spatial autocorrelation (random distribution of spatial variance) and 1 representing significant positive spatial autocorrelation (specific distribution of spatial variance). When we compare the ranks of the observed coefficient against the transformed raw p-values (Response Fig. 3B), the v-shape shows that both positive and negative spatial autocorrelation coefficients can be statistically significant. To illustrate the spatial expression pattern of representative genes that have negative, neutral and positive autocorrelation, we visualise the gene expression of Sntg1, Tnrc6b and Ttr (Response Fig. 3C). We see that Ttr, a gene with a significant p-value and a positive autocorrelation, aligns with what we define as a spatially variable gene. Therefore, we believe that not all methods can be ranked by raw p-values, as in this case, we would be prioritizing genes that are not spatially variable.*

**Response Figure 1.** Concordance of SVG rankings reported from each SVG method compared in a pairwise fashion using Spearman’s correlation coefficient. (A) original, and (B) Spearman’s correlation calculated using -log10 transformed raw p-values for all applicable methods.

**Response Figure 2.** Scatter plots of the ranked transformed raw p-values against the ranked BH-adjusted p-values for Giotto KM and Moran’s I, and the ranked likelihood ratio (LLR) statistic for SOMDE. Two example datasets Vickovic et al. D2 and Wu et al. D1 are shown.

**Response Figure 3.** (A) Schematic illustrating relationships between Moran’s I statistics (ranging from -1 to 1) and p-values. (B) Scatter plots of comparisons between ranked Moran’s I gene statistics and ranked raw and adjusted -log10 transformed p-values for the Vickovic et al. D2 dataset. Three genes representative of negative spatial autocorrelation (close to -1, Sntg1), no autocorrelation (close to 0, Tnrc6b) and positive autocorrelation (close to 1, Ttr) are highlighted. (C) Visualisation of the log expression of the representative genes across the spots on the tissue.

2. The manuscript extended the downsampling experiment from one dataset to two datasets, but we don’t know if the conclusions are generalizable. It would provide readers with a comprehensive comparison to perform the analysis across all datasets used in the study and summarize the results.

***Response:*** *We appreciate this suggestion. We have now extended both downsampling analyses to include all datasets used in our benchmarking study summarised as boxplot visualisations shown in Response Fig. 4 and have included this figure (Figure 4) in our updated manuscript accordingly (see section “Dependency of SVG statistics across genes and spatial spots”).*

**Response Figure 4.** (a) Schematic of the experimental approach to determine the interdependency of genes in the calculation of SVG statistics. For the down-sampling, 50% of the genes were randomly down-sampled whilst keeping the number of spots equal. (b) Boxplots of the Spearman correlation results performed on all datasets coloured by SVG method and ordered by increasing mean correlation coefficient. (c-d) as in (a-b) but for the investigation of the reproducibility of SVG methods with increased sparsity in spatial spots. The datasets were randomly down sampled to 80% of the total number of spots. (e) Venn diagram illustrates the proportions of uniquely identified SVGs as described below in (f). Boxplots of the proportion of false positive SVGs calculated as the proportion of SVGs uniquely identified in the subsampled data divided by all the significant SVGs identified in the subsampled data.

**Third round of review**

**Reviewer 1**

All my previous comments has been addressed.

**Reviewer 2**

The revised manuscript has addressed my remaining questions.
